# Ethyl 2-[(*tert*-but­oxy­carbon­yl)amino]­thia­zole-5-carboxyl­ate

**DOI:** 10.1107/S1600536812005971

**Published:** 2012-02-17

**Authors:** Weisong Wang, Bohua Zhong, Weiguo Shi

**Affiliations:** a Beijing Institute of Pharmacology and Toxicology, Beijing 100850, People’s Republic of China

## Abstract

In the crystal of the title compound, C_11_H_16_N_2_O_4_S, molecules are linked *via* pairs of N—H⋯N hydrogen bonds to form inversion dimers. The dimers are linked by a weak C—H⋯O interaction to form chains propagating along direction [100].

## Related literature

For details of the synthesis, see: Upadhyaya *et al.* (2007 ▶). For the bioactivity of thia­zoles, see: Barradas *et al.* (2011 ▶); Zaharia *et al.* (2010 ▶). For related structures, see: Liu *et al.* (2011 ▶); Wang (2011 ▶).
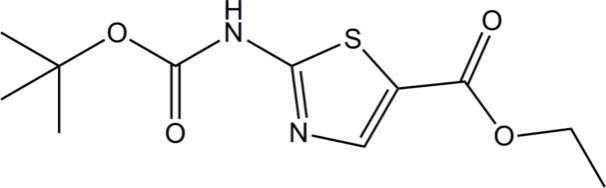


## Experimental

### 

#### Crystal data

C_11_H_16_N_2_O_4_S
*M* *_r_* = 272.32
Monoclinic, 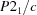

*a* = 5.8258 (12) Å
*b* = 9.4916 (19) Å
*c* = 24.350 (5) Å
β = 92.37 (3)°
*V* = 1345.3 (5) Å^3^
*Z* = 4
Mo *K*α radiation
μ = 0.25 mm^−1^
*T* = 113 K
0.26 × 0.24 × 0.22 mm

#### Data collection

Rigaku Saturn CCD diffractometer
Absorption correction: multi-scan *CrystalClear* (Rigaku, 2005 ▶) *T*_min_ = 0.938, *T*_max_ = 0.947
11392 measured reflections
3180 independent reflections
2177 reflections with *I* > 2σ(*I*)
*R*_int_ = 0.057

#### Refinement

*R*[*F* ^2^ > 2σ(*F* ^2^)] = 0.054
*wR*(*F* ^2^) = 0.155
*S* = 1.07
3180 reflections
172 parameters
H atoms treated by a mixture of independent and constrained refinement
Δρ_max_ = 0.46 e Å^−3^
Δρ_min_ = −0.46 e Å^−3^

### 

Data collection: *CrystalClear* (Rigaku, 2005 ▶); cell refinement: *CrystalClear*; data reduction: *CrystalClear*; program(s) used to solve structure: *SHELXS97* (Sheldrick, 2008 ▶); program(s) used to refine structure: *SHELXL97* (Sheldrick, 2008 ▶); molecular graphics: *SHELXTL* (Sheldrick, 2008 ▶); software used to prepare material for publication: *CrystalStructure* (Rigaku, 2005 ▶).

## Supplementary Material

Crystal structure: contains datablock(s) I, global. DOI: 10.1107/S1600536812005971/ff2055sup1.cif

Structure factors: contains datablock(s) I. DOI: 10.1107/S1600536812005971/ff2055Isup4.hkl

Supplementary material file. DOI: 10.1107/S1600536812005971/ff2055Isup3.cml

Additional supplementary materials:  crystallographic information; 3D view; checkCIF report

## Figures and Tables

**Table 1:** Hydrogen-bond geometry (Å, °)

*D*—H⋯*A*	*D*—H	H⋯*A*	*D*⋯*A*	*D*—H⋯*A*
N2—H2*A*⋯N1^i^	0.84 (3)	2.01 (3)	2.844 (3)	172 (3)
C10—H10*B*⋯O1^ii^	0.98	2.54	3.418 (3)	149

Symmetry codes: (i) 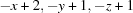; (ii) 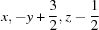.

**Fig. 1.**
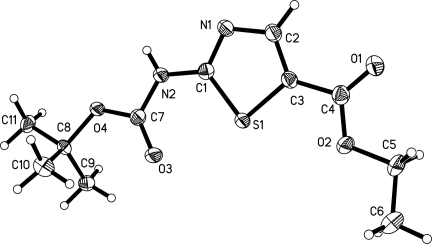
Structure of the title compound, with displacement ellipsoids drawn at 50% probability level.

**Fig. 2.**
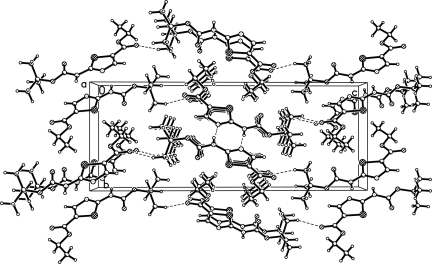
Part of the packing of the title compound, viewed down the a direction. Hydrogen bonds are shown as dashed lines.

## supplementary crystallographic information

### Experimental

Ethyl 2-aminothiazole-5-carboxylate 5 g (Alfa Aesar) was dissolved in 30 ml 1,4-dioxane, then triethylamine 3 ml and di-*tert*-butyl carbonate 6 g were added into the solution, the reaction mixture was stirred for 6 h at room temperature. When the reaction was complete as shown by TLC, the solvent was removed under reduced pressure. The residue was added into 200 ml water, ethyl acetate 100 ml was then added into the solution, the mixture was stirred for 10 min. The organic layer was separated and washed by water, brine, and dried over Na_2_SO_4_. The solvent was removed under reduced pressure to yield product as a white solid (4.7 g, 59.5%).

### Refinement

The H atoms linked to the C atoms were fixed geometrically and treated as riding with C—H = 0.95 Å (aromatic), 0.98 Å (ethyl), 0.99 Å (methylene) with *U*_iso_(H) = 1.2–1.5Ueq(C).

### Crystal data

**Table:**

C_11_H_16_N_2_O_4_S	*F*(000) = 576
*M**_r_* = 272.32	*D*_x_ = 1.345 Mg m^−^^3^
Monoclinic, *P*2_1_/*c*	Mo *K*α radiation, λ = 0.71073 Å
*a* = 5.8258 (12) Å	Cell parameters from 2589 reflections
*b* = 9.4916 (19) Å	θ = 2.3–28.0°
*c* = 24.350 (5) Å	µ = 0.25 mm^−^^1^
β = 92.37 (3)°	*T* = 113 K
*V* = 1345.3 (5) Å^3^	Block, colourless
*Z* = 4	0.26 × 0.24 × 0.22 mm

### Data collection

**Table:**

Rigaku Saturn CCD diffractometer	3180 independent reflections
Radiation source: rotating anode	2177 reflections with *I* > 2σ(*I*)
Confocal monochromator	*R*_int_ = 0.057
Detector resolution: 7.31 pixels mm^-1^	θ_max_ = 28.0°, θ_min_ = 2.3°
ω and φ scans	*h* = −7→7
Absorption correction: multi-scan *CrystalClear* (Rigaku, 2005)	*k* = −12→12
*T*_min_ = 0.938, *T*_max_ = 0.947	*l* = −32→30
11392 measured reflections	

### Refinement

**Table:**

Refinement on *F*^2^	Secondary atom site location: difference Fourier map
Least-squares matrix: full	Hydrogen site location: inferred from neighbouring sites
*R*[*F*^2^ > 2σ(*F*^2^)] = 0.054	H atoms treated by a mixture of independent and constrained refinement
*wR*(*F*^2^) = 0.155	*w* = 1/[σ^2^(*F*_o_^2^) + (0.0806*P*)^2^] where *P* = (*F*_o_^2^ + 2*F*_c_^2^)/3
*S* = 1.07	(Δ/σ)_max_ < 0.001
3180 reflections	Δρ_max_ = 0.46 e Å^−^^3^
172 parameters	Δρ_min_ = −0.46 e Å^−^^3^
0 restraints	Extinction correction: *SHELXL97* (Sheldrick, 2008), Fc^*^=kFc[1+0.001xFc^2^λ^3^/sin(2θ)]^-1/4^
Primary atom site location: structure-invariant direct methods	Extinction coefficient: 0.014 (4)

### Special details

**Table:**

Geometry. All e.s.d.'s (except the e.s.d. in the dihedral angle between two l.s. planes) are estimated using the full covariance matrix. The cell e.s.d.'s are taken into account individually in the estimation of e.s.d.'s in distances, angles and torsion angles; correlations between e.s.d.'s in cell parameters are only used when they are defined by crystal symmetry. An approximate (isotropic) treatment of cell e.s.d.'s is used for estimating e.s.d.'s involving l.s. planes.
Refinement. Refinement of *F*^2^ against ALL reflections. The weighted *R*-factor *wR* and goodness of fit *S* are based on *F*^2^, conventional *R*-factors *R* are based on *F*, with *F* set to zero for negative *F*^2^. The threshold expression of *F*^2^ > σ(*F*^2^) is used only for calculating *R*-factors(gt) *etc*. and is not relevant to the choice of reflections for refinement. *R*-factors based on *F*^2^ are statistically about twice as large as those based on *F*, and *R*-factors based on ALL data will be even larger.

### Fractional atomic coordinates and isotropic or equivalent isotropic displacement parameters (Å^2^)

**Table:**

	*x*	*y*	*z*	*U*_iso_*/*U*_eq_	
S1	0.48801 (9)	0.74851 (5)	0.499500 (19)	0.0218 (2)	
O1	0.4912 (3)	0.86694 (18)	0.65493 (6)	0.0358 (4)	
O2	0.2511 (3)	0.91136 (16)	0.58203 (6)	0.0277 (4)	
O3	0.5169 (3)	0.68642 (17)	0.39011 (6)	0.0287 (4)	
O4	0.7506 (3)	0.51367 (14)	0.35826 (6)	0.0211 (4)	
N1	0.8382 (3)	0.60995 (19)	0.54014 (7)	0.0240 (4)	
N2	0.7723 (3)	0.5693 (2)	0.44654 (7)	0.0229 (4)	
C1	0.7172 (4)	0.6345 (2)	0.49427 (9)	0.0211 (5)	
C2	0.7472 (4)	0.6836 (2)	0.58220 (9)	0.0251 (5)	
H2	0.8121	0.6796	0.6186	0.030*	
C3	0.5600 (4)	0.7625 (2)	0.56921 (8)	0.0217 (5)	
C4	0.4342 (4)	0.8514 (2)	0.60725 (9)	0.0257 (5)	
C5	0.1173 (5)	1.0034 (3)	0.61617 (11)	0.0347 (6)	
H5A	0.2188	1.0727	0.6355	0.042*	
H5B	0.0377	0.9476	0.6440	0.042*	
C6	−0.0552 (4)	1.0780 (3)	0.57876 (11)	0.0353 (6)	
H6A	0.0257	1.1341	0.5518	0.053*	
H6B	−0.1502	1.1402	0.6005	0.053*	
H6C	−0.1532	1.0084	0.5595	0.053*	
C7	0.6642 (4)	0.5978 (2)	0.39667 (9)	0.0222 (5)	
C8	0.6805 (4)	0.5341 (2)	0.29934 (8)	0.0203 (5)	
C9	0.4230 (4)	0.5149 (2)	0.29012 (10)	0.0253 (5)	
H9A	0.3779	0.4226	0.3042	0.038*	
H9B	0.3828	0.5204	0.2507	0.038*	
H9C	0.3423	0.5892	0.3095	0.038*	
C10	0.7627 (4)	0.6774 (2)	0.28086 (10)	0.0295 (5)	
H10A	0.6756	0.7513	0.2989	0.044*	
H10B	0.7391	0.6857	0.2409	0.044*	
H10C	0.9265	0.6879	0.2909	0.044*	
C11	0.8063 (4)	0.4163 (2)	0.27124 (9)	0.0257 (5)	
H11A	0.9718	0.4255	0.2793	0.039*	
H11B	0.7752	0.4220	0.2314	0.039*	
H11C	0.7531	0.3252	0.2848	0.039*	
H2A	0.881 (5)	0.512 (3)	0.4476 (13)	0.047 (9)*	

### Atomic displacement parameters (Å^2^)

**Table:**

	*U*^11^	*U*^22^	*U*^33^	*U*^12^	*U*^13^	*U*^23^
S1	0.0254 (3)	0.0232 (3)	0.0166 (3)	0.0059 (2)	−0.0008 (2)	−0.0012 (2)
O1	0.0432 (11)	0.0404 (10)	0.0237 (9)	0.0136 (8)	−0.0007 (7)	−0.0049 (8)
O2	0.0284 (9)	0.0279 (9)	0.0267 (9)	0.0098 (7)	−0.0011 (6)	−0.0062 (7)
O3	0.0319 (9)	0.0309 (9)	0.0231 (8)	0.0136 (7)	−0.0018 (6)	−0.0013 (7)
O4	0.0237 (8)	0.0235 (8)	0.0161 (8)	0.0064 (6)	−0.0018 (6)	−0.0022 (6)
N1	0.0288 (10)	0.0242 (10)	0.0188 (9)	0.0043 (8)	−0.0004 (7)	0.0005 (7)
N2	0.0262 (10)	0.0241 (10)	0.0182 (9)	0.0082 (8)	−0.0007 (7)	−0.0010 (7)
C1	0.0236 (11)	0.0178 (11)	0.0219 (11)	0.0005 (9)	0.0019 (8)	−0.0002 (8)
C2	0.0302 (12)	0.0247 (12)	0.0202 (11)	0.0027 (9)	0.0010 (8)	−0.0003 (9)
C3	0.0257 (11)	0.0208 (11)	0.0185 (10)	0.0001 (9)	−0.0005 (8)	−0.0014 (8)
C4	0.0296 (12)	0.0230 (12)	0.0246 (12)	0.0013 (10)	0.0033 (9)	−0.0007 (9)
C5	0.0353 (14)	0.0347 (14)	0.0342 (15)	0.0132 (11)	0.0041 (11)	−0.0099 (11)
C6	0.0294 (13)	0.0291 (13)	0.0478 (16)	0.0058 (10)	0.0052 (11)	−0.0024 (12)
C7	0.0241 (11)	0.0204 (11)	0.0221 (11)	0.0022 (9)	0.0018 (8)	−0.0011 (9)
C8	0.0221 (11)	0.0246 (11)	0.0140 (10)	0.0005 (9)	−0.0005 (8)	−0.0004 (8)
C9	0.0205 (11)	0.0318 (13)	0.0233 (12)	−0.0004 (9)	−0.0018 (9)	0.0000 (9)
C10	0.0326 (13)	0.0258 (13)	0.0299 (12)	−0.0068 (10)	−0.0027 (9)	0.0015 (10)
C11	0.0231 (12)	0.0313 (13)	0.0228 (11)	0.0028 (9)	0.0029 (8)	−0.0080 (9)

### Geometric parameters (Å, º)

**Table:**

S1—C1	1.727 (2)	C5—H5A	0.9900
S1—C3	1.737 (2)	C5—H5B	0.9900
O1—C4	1.204 (3)	C6—H6A	0.9800
O2—C4	1.336 (3)	C6—H6B	0.9800
O2—C5	1.455 (3)	C6—H6C	0.9800
O3—C7	1.208 (2)	C8—C11	1.515 (3)
O4—C7	1.343 (2)	C8—C10	1.517 (3)
O4—C8	1.488 (2)	C8—C9	1.518 (3)
N1—C1	1.317 (3)	C9—H9A	0.9800
N1—C2	1.365 (3)	C9—H9B	0.9800
N2—C1	1.367 (3)	C9—H9C	0.9800
N2—C7	1.371 (3)	C10—H10A	0.9800
N2—H2A	0.84 (3)	C10—H10B	0.9800
C2—C3	1.349 (3)	C10—H10C	0.9800
C2—H2	0.9500	C11—H11A	0.9800
C3—C4	1.471 (3)	C11—H11B	0.9800
C5—C6	1.505 (4)	C11—H11C	0.9800
			
C1—S1—C3	87.93 (10)	H6A—C6—H6C	109.5
C4—O2—C5	115.48 (18)	H6B—C6—H6C	109.5
C7—O4—C8	119.88 (16)	O3—C7—O4	127.3 (2)
C1—N1—C2	109.57 (18)	O3—C7—N2	123.5 (2)
C1—N2—C7	123.24 (19)	O4—C7—N2	109.13 (18)
C1—N2—H2A	118 (2)	O4—C8—C11	102.75 (16)
C7—N2—H2A	119 (2)	O4—C8—C10	108.99 (17)
N1—C1—N2	120.32 (19)	C11—C8—C10	111.31 (18)
N1—C1—S1	115.88 (16)	O4—C8—C9	110.91 (17)
N2—C1—S1	123.78 (16)	C11—C8—C9	109.73 (18)
C3—C2—N1	116.31 (19)	C10—C8—C9	112.70 (18)
C3—C2—H2	121.8	C8—C9—H9A	109.5
N1—C2—H2	121.8	C8—C9—H9B	109.5
C2—C3—C4	126.1 (2)	H9A—C9—H9B	109.5
C2—C3—S1	110.30 (16)	C8—C9—H9C	109.5
C4—C3—S1	123.57 (16)	H9A—C9—H9C	109.5
O1—C4—O2	125.0 (2)	H9B—C9—H9C	109.5
O1—C4—C3	123.6 (2)	C8—C10—H10A	109.5
O2—C4—C3	111.36 (19)	C8—C10—H10B	109.5
O2—C5—C6	107.3 (2)	H10A—C10—H10B	109.5
O2—C5—H5A	110.3	C8—C10—H10C	109.5
C6—C5—H5A	110.3	H10A—C10—H10C	109.5
O2—C5—H5B	110.3	H10B—C10—H10C	109.5
C6—C5—H5B	110.3	C8—C11—H11A	109.5
H5A—C5—H5B	108.5	C8—C11—H11B	109.5
C5—C6—H6A	109.5	H11A—C11—H11B	109.5
C5—C6—H6B	109.5	C8—C11—H11C	109.5
H6A—C6—H6B	109.5	H11A—C11—H11C	109.5
C5—C6—H6C	109.5	H11B—C11—H11C	109.5
			
C2—N1—C1—N2	−178.4 (2)	C2—C3—C4—O1	2.5 (4)
C2—N1—C1—S1	0.3 (2)	S1—C3—C4—O1	−175.89 (19)
C7—N2—C1—N1	−175.1 (2)	C2—C3—C4—O2	−177.5 (2)
C7—N2—C1—S1	6.3 (3)	S1—C3—C4—O2	4.1 (3)
C3—S1—C1—N1	−0.62 (17)	C4—O2—C5—C6	170.96 (19)
C3—S1—C1—N2	178.0 (2)	C8—O4—C7—O3	6.0 (3)
C1—N1—C2—C3	0.3 (3)	C8—O4—C7—N2	−173.39 (17)
N1—C2—C3—C4	−179.4 (2)	C1—N2—C7—O3	2.8 (4)
N1—C2—C3—S1	−0.8 (3)	C1—N2—C7—O4	−177.82 (18)
C1—S1—C3—C2	0.74 (17)	C7—O4—C8—C11	−177.64 (17)
C1—S1—C3—C4	179.4 (2)	C7—O4—C8—C10	64.2 (2)
C5—O2—C4—O1	1.0 (3)	C7—O4—C8—C9	−60.4 (2)
C5—O2—C4—C3	−179.05 (19)		

### Hydrogen-bond geometry (Å, º)

**Table:**

*D*—H···*A*	*D*—H	H···*A*	*D*···*A*	*D*—H···*A*
N2—H2*A*···N1^i^	0.84 (3)	2.01 (3)	2.844 (3)	172 (3)
C10—H10*B*···O1^ii^	0.98	2.54	3.418 (3)	149

Symmetry codes: (i) −*x*+2, −*y*+1, −*z*+1; (ii) *x*, −*y*+3/2, *z*−1/2.
